# Cellular liquid biopsy provides unique chances for disease monitoring, preclinical model generation and therapy adjustment in rare salivary gland cancer patients

**DOI:** 10.1002/1878-0261.13741

**Published:** 2024-10-05

**Authors:** Nataša Stojanović Gužvić, Florian Lüke, Steffi Treitschke, Andrea Coluccio, Martin Hoffmann, Giancarlo Feliciello, Adithi Ravikumar Varadarajan, Xin Lu, Kathrin Weidele, Catherine Botteron, Silvia Materna–Reichelt, Felix Keil, Katja Evert, Florian Weber, Thomas Schamberger, Michael Althammer, Jirka Grosse, Dirk Hellwig, Christian Schulz, Stephan Seitz, Peter Ugocsai, Anke Schlenska‐Lange, Roman Mayr, Ulrich Kaiser, Wolfgang Dietmaier, Bernhard Polzer, Jens Warfsmann, Kamran Honarnejad, Tobias Pukrop, Daniel Heudobler, Christoph A. Klein, Christian Werno

**Affiliations:** ^1^ Fraunhofer Institute for Toxicology and Experimental Medicine ITEM‐R Germany; ^2^ Department of Internal Medicine III University Hospital Regensburg Germany; ^3^ Bavarian Cancer Research Center (BZKF) Regensburg Germany; ^4^ Insitute for Pathology University of Regensburg Germany; ^5^ Experimental Medicine and Therapy Research University of Regensburg Germany; ^6^ Department of Nuclear Medicine University Hospital Regensburg Germany; ^7^ Department of Internal Medicine II University Hospital Regensburg Germany; ^8^ Department of Obstetrics and Gynecology University Hospital Regensburg Germany; ^9^ Department of Oncology and Hematology Hospital Barmherzige Brüder Regensburg Germany; ^10^ Department of Urology, Caritas St. Josef Medical Center University of Regensburg Germany

**Keywords:** CTC, liquid biopsy, personalized tumor therapy, salivary gland cancer, tumoroid culture

## Abstract

While cell‐free liquid biopsy (cfLB) approaches provide simple and inexpensive disease monitoring, cell‐based liquid biopsy (cLB) may enable additional molecular genetic assessment of systemic disease heterogeneity and preclinical model development. We investigated 71 blood samples of 62 patients with various advanced cancer types and subjected enriched circulating tumor cells (CTCs) to organoid culture conditions. CTC‐derived tumoroid models were characterized by DNA/RNA sequencing and immunohistochemistry, as well as functional drug testing. Results were linked to molecular features of primary tumors, metastases, and CTCs; CTC enumeration was linked to disease progression. Of 52 samples with positive CTC counts (≥1) from eight different cancer types, only CTCs from two salivary gland cancer (SGC) patients formed tumoroid cultures (*P* = 0.0005). Longitudinal CTC enumeration of one SGC patient closely reflected disease progression during treatment and revealed metastatic relapse earlier than clinical imaging. Multiomics analysis and functional *in vitro* drug testing identified potential resistance mechanisms and drug vulnerabilities. We conclude that cLB might add a functional dimension (to the genetic approaches) in the personalized management of rare, difficult‐to‐treat cancers such as SGC.

## Graphical abstract

We quantified and cultured circulating tumor cells (CTCs) of 62 patients with various cancer types and generated CTC‐derived tumoroid models from two salivary gland cancer patients. Cellular liquid biopsy‐derived information enabled molecular genetic assessment of systemic disease heterogeneity and functional testing for therapy selection in both salivary gland cancer patients, which may provide a paradigm for other rare cancers.

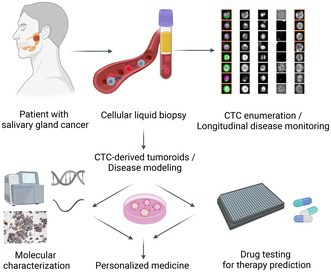

**Abbreviations**
AdCC: adenoid cystic carcinoma
AR: androgen receptor
cfLB: cell‐free liquid biopsy
cLB: cellular liquid biopsy
CTC: circulating tumor cell
CNA: copy number alteration
EpCAM: epithelial cell adhesion molecule
EGFR: epithelial growth factor receptor
FDG PET/CT: fluorodeoxyglucose positron emission tomography/computerized tomography
FFPE: formalin‐fixed, paraffin‐embedded
GSEA: gene set enrichment analysis
HE: hematoxylin eosin
IHC: immunohistochemistry
KEGG: Kyoto Encyclopedia of Genes and Genomes
LN: lymph node
m: month
PT: primary tumor
SCNEC: small cell neuroendocrine carcinoma
SCLC: small cell lung cancer
SGC: salivary gland cancer
SUV: standardized uptake value
WGA: whole genome amplification
WGS: whole genome sequencing
WTA: whole transcriptome amplification

## 1 Introduction

Great optimism rests on the use of blood‐derived liquid biopsy (LB) approaches for better monitoring of disease courses, informed therapy adjustment, and consequently long‐term disease control. Cell‐free (cf)LB studies of proteins, lipids, extracellular vesicles, coding and non‐coding RNA species, and DNA are all under intensive clinical‐translational scrutiny [1, 2, 3, 4, 5, 6]. In comparison, cellular (c)LB has lost ground due to its higher cost and lower level of automation, although its clinical utility has been well documented for a variety of clinical settings [7]. However, using cLB provides additional information regarding: (i) the assessment of cellular heterogeneity, (ii) detection of underrepresented pre‐existing therapy escape variants, (iii) perspective of combined assessment of regulatory mechanisms (i.e., combined genome and transcriptome analysis; combined genomic and epigenomic analysis; etc.), and (iv) the chance of generating pre‐clinical models for therapy‐accompanying drug testing.

So far, few studies reported on successful circulating tumor cell (CTC) expansion *in vivo* or *in vitro*. After an initial landmark study [8] generating xenografts from breast cancer patients with very high CTC counts, follow‐up studies in mice or 2D or 3D *in vitro* cultures resulted in CTC‐derived models of melanoma, colorectal, pancreatic, lung, breast, gastroesophageal, and prostate cancer patients [9]. However, although more than 50 CTC‐derived models have been described until now, CTC model generation is mostly limited to aggressive cancer types such as small cell lung cancer (SCLC) or melanoma. Therefore, due to the low frequency of CTCs and the lack of optimized culturing conditions the success rate for the generation of CTC‐derived models has been relatively low while the time span to expand the cells has been rather long [9].

On the other hand, more and more studies indicate that drug testing on primary tumor (PT) tissue‐derived preclinical *in vivo* and *in vitro* models recapitulates individual therapy responses of patients and therefore may improve precision cancer care [10, 11, 12, 13]. As the establishment of preclinical models is mainly limited by the availability of viable tumor tissue [12], successful model generation derived from CTCs is desirable to perform personalized drug screening or uncover resistance mechanisms.

We therefore asked whether CTCs from cancers other than those described above can be expanded in culture with a high success rate and tested several cancer types applying widely used conditions for organoid generation. For such cancers, we additionally aimed to examine whether CTC numbers reflect disease dynamics. Molecular genetic analysis of established models with *ex vivo* derived information should provide insight as to what extent such models could complement routine clinical information for decision making.

## 2 Materials and methods

### 2.1 Patients

Sixty‐two patients with various advanced cancer types were recruited from 2018 to 2021 at the University Hospital (Regensburg), the Hospital Barmherzige Brüder (Regensburg), and the Caritas St. Josef Medical Center (Regensburg). Two patients with SGC were treated at the University Hospital Regensburg in 2019 and 2020 and we obtained clinical, laboratory, and outcome data from each patient's medical records. Clinical outcomes were followed up until the death of both patients. The study was approved by the ethics committee of the University of Regensburg (ethics statement No.: 07‐079; 18‐948‐101 and 17‐672‐101) and written informed consent was obtained from all patients. The study was performed in accordance with the Declaration of Helsinki.

### 2.2 Blood collection, CTC enumeration, enrichment, and isolation

Blood was drawn under sterile conditions according to our institutional standards. In SGC patient No.1 central vein blood was drawn using a port catheter system. Eight longitudinal blood draws were performed between 14.5 and 26 months from the diagnosis. In SGC patient No.2 blood was drawn from a peripheral arm vein.

For CTC enumeration, 7.5 mL of blood was collected in CellSave Preservative Tubes and analyzed with CellSearch® system (Menarini Silicon Biosystems, Bologna, Italy) within 72 h after blood draw, using CellSearch® Circulating Tumor Cell Kit and CellSearch® Tumor Phenotyping Reagent HER/2neu (Menarini Silicon Biosystems). CTCs (CD45‐, cytokeratins 8, 18+, and/or 19+ and Her2+/− cells) were transferred on chamber slides (Nunc; Thermo Fisher Scientific, Waltham, MA, USA) and were manually isolated using a micromanipulator (Eppendorf, Hamburg, Germany) and downstream molecular analysis were performed. For isolation of viable cells, 8.5–26 mL of blood were collected in BD Vacutainer® K2EDTA – tubes (Thermo Fisher Scientific). For negative selection, CTCs were enriched by using RosetteSep™ CTC Enrichment Cocktail Containing Anti CD56 Kit and for small cell lung cancer samples, RosetteSep™ CTC Enrichment Cocktail Containing Anti CD36 Kit (Stem Cells Technologies, Cologne, Germany) by following manufacturer's instructions. For density separation, Percoll 50% (Percoll‐100% diluted with sodium chloride) was used. Nine milliliter of whole blood was diluted 1:2 with PBS containing 2% FCS and was carefully loaded on equal amount (18 mL) of Percoll 50% in a 50 mL tube. The sample was centrifuged for 20 min at 1000 g at room temperature (RT) and brake set on 1. After centrifugation, the interphase layer was collected in the new tube and washed twice with PBS containing 2% FCS by using 300 ***g*** centrifugal force for 5 min.

### 2.3 [ ^18^F]FDG PET/CT and image analysis

[^18^F]FDG PET/CT imaging was performed on two SGC patients using a Biograph mCT 40 PET/CT scanner for the first and second scan and a Biograph 16 PET/CT scanner for the third to fifth scan (both Siemens Healthineers, Erlangen, Germany). After overnight fasting, 3 MBq [^18^F]FDG per kilogram body weight were injected intravenously (290 ± 10 MBq). The patients' blood glucose level was 142 ± 18 mg⋅dL^−1^ (7.88 ± 1.00 mmol⋅L^−1^). To minimize muscular [^18^F]FDG uptake patients were advised to stay in a quiet lying position. Warming blankets were used to keep the patient warm and minimize possible accumulation of the tracer in the brown adipose tissue. Prior to scanning all metal parts were removed and the patient was instructed to void the bladder.

After a waiting period of about 60 min post‐injection (59 ± 6 min), PET/CT acquisition was performed with elevated arms to acquire images of the head and trunk (first, third, and fourth scan) or with adducted arms (second and fifth scan). The same area was covered by a low‐dose CT scan (tube current <50 mAs, tube voltage 120 kV) if no contrast agents were used (first, second, and third scan). Otherwise, 130 mL of Accupaque™ 300 (GE Healthcare, Braunschweig, Germany) was applied as an intravenous contrast agent with consecutive full‐dose CT acquisition (120 kV, <100 mAs) for the fourth and fifth scans. PET images (slice thickness 5 mm) were corrected for random coincidences, decay, scatter, and attenuation and reconstructed iteratively using vendor parameter presets. PET and CT images were checked for breathing and motion artifacts and scaled to allow measurement of the standardized uptake value (SUV). Images were displayed using syngo.via version VB40 (Siemens Healthineers, Erlangen, Germany) and interpreted by two experienced nuclear medicine physicians with reference to maximum intensity projection, PET/CT fusion, and CT images, until consensus was reached. The observers were blinded to clinical parameters and patient outcome.

### 2.4 Standard cell culture

Working with human cell lines and tumoroid cultures was performed under sterile conditions. Commercially available cell lines MCF‐7 (RRID:CVCL_0031) were obtained from the German Collection of Microorganisms and Cell Cultures (DSMZ, Braunschweig, Germany) and BT‐474 (RRID:CVCL_0179) from ATCC (Manassas, VA, USA). Cells were cultured in RPMI (MCF‐7) or DMEM medium (BT‐474) supplemented with 10% FCS, 1× GlutaMax, 1× PenStrep. In the case of MCF7 cell line, the medium was additionally supplemented with 0.01 mg⋅mL^−1^ insulin. The cells were further cultured in tumoroid conditions as described for CTC generated tumoroid models below. The identity of all cell lines was confirmed by DNA fingerprinting analysis utilizing the GenePrint® 10 System (Promega, Walldorf, Germany). All experiments were performed with mycoplasma‐free cells.

### 2.5 Cultivation of CTCs as tumoroids

Cells pelleted after negative selection or gradient enrichment were resuspended in Geltrex™ LDEV‐free Reduced Growth Factor Basement Membrane Matrix, seeded in 30 μL domes in 48 well plate, and incubated at 37 °C for 30 min enabling Geltrex™ to polymerase. Domes were then covered with 400 μL tumoroid culture medium [14] which was replaced every 3–4 days. In the case of prostate cancer samples, the medium was supplemented with 1 nm prostaglandin and 1 nm dihydrotestosterone (DHT) and in the case of gastric cancer, with 1 nm gastrin. Domes were screened for tumoroid growth once per week and when tumoroids reached a diameter of 70–150 μm, they were passaged by using mechanical force and enzymatic approach (TrypLE™ Express Enzyme; ThermoFisher Scientific, Waltham, MA, USA) in 1:2 or 1:3 ratio. Intact tumoroids needed for immunochistochemical (IHC) analysis, whole genome amplification (WGA), and RNA sequencing (RNA‐Seq) were harvested by using Cultrex Organoid Harvesting Solution according to the manufacturer instructions (Bio‐Techne TrevigenBio, Minneapolis, MN, USA). In order to avoid attachment of tumoroids to the pipette tip or tube, these had to be precoated with 0.5% BSA. Single tumoroids with maximal size of 50 μm or pools of 5–10 tumoroids, were manually picked in the same way as single CTCs, as described above.

### 2.6 Genomic DNA isolation from bulk tumoroids and FFPE tissue

Bulk genomic DNA from tumoroids was isolated from single cell suspension of separated tumoroids using DNeasy Blood & Tissue Kit (Qiagen, Hilden, Germany). DNA from formalin‐fixed, paraffin‐embedded (FFPE) tissue was isolated from tumor punches or 10–20 μm slices using QIAamp DNA FFPE Tissue Kit (Qiagen). Areas with high tumor content were previously marked by an experienced pathologist.

### 2.7 Whole genome amplification of CTCs, single tumoroids, and DNA from FFPE samples

After harvest, cells were collected and transferred on chamber slides (Nunc; Thermo Fisher Scientific), enabling manual isolation of single cells and cell pools using a micromanipulator (Eppendorf, Hamburg, Germany). For further molecular downstream analysis, isolated single cells (from CellSearch system but also from cultured tumoroids) were subjected to simultaneous whole transcriptome amplification (WTA) and whole genome amplification (WGA) as previously described [15, 16, 17].

The quality of WGA and WTA products was assessed using multiplex PCR assays (Ampli1™ QC Kit and Ampli 1™ WTA QC Kit; Menarini Silicon Biosystems).

Isolated CTCs, CTC‐derived tumoroids, and genomic DNA from FFPE samples (primary tumor (PT) and metastasis) were subjected to WGA [15, 16]. The quality of WGA products was assessed using multiplex PCR assay as previously described [17]. WGA with sufficient quality (genome integrity index (GII) > 1) were either directly used or re‐amplified and prepared for further downstream analyses.

### 2.8 LowPass whole genome sequencing (WGS) and copy number alteration (CNA) profiling

Libraries for low‐pass genome sequencing were prepared using Ampli1™ LowPass kit (Menarini Silicon Biosystems) for Illumina® platforms (Illumina®, San Diego, CA, USA). Briefly, 5 μL of the original Ampli1™ WGA product (in case of CTC‐derived tumoroids and FFPE samples) or reamplified WGA (in case of CTCs detected by CellSearch device) have been purified with 1.8× volume of SPRI beads (Beckman Coulter, Krefeld, Germany) and eluted in 22 μL of nuclease‐free pure water. Three microliters of purified products have been subsequently used to prepare barcoded libraries  according to the instructions from the manufacturer. The libraries were quantified using Qubit dsDNA HS Assay kit and Qubit 2.0 Fluorometer (Thermo Fisher Scientific). Additionally, the average fragment sizes of the libraries were assessed using the Agilent High Sensitivity DNA Kit on the Agilent 2100 Bioanalyzer System (Agilent Technologies, Waldbronn, Germany). A maximum number of 30 barcoded libraries were mixed in equimolar concentrations to obtain a 4 nm pool ready for sequencing. Ampli1™ LowPass libraries sequencing was performed in single read (SR) mode on a MiSeq System (Illumina®) with MiSeq Reagent Kit v3 (150‐cycle) (Illumina®).

The bioinformatics CNA‐profile analysis started, after de‐multiplexing, with raw FASTQ files, which were submitted to the LowPass workflow from our in‐house NGS analysis collection HIENA 0.9.7.0. The raw sequence data from 50 samples were trimmed with BBDuk 38.84 [18], removing adapter sequences and poor‐quality bases at the end of the forward reads. Non‐human reads originating from microbial and/or fungal contamination of fresh water or reagents might interfere with downstream analysis. Therefore, read decontamination was performed using BioBloom Tools 2.0.13 [19] with filters for the genomes of *Homo sapiens* (hg38), *Mus musculus* (mm38), *Escherichia coli* (BL21), *Mycoplasma pneumoniae* (M129), *Sphingobium* sp. (SYK‐6), *Bradyrhizobium japonicum* (USDA 110), *Pichia pastoris* (GS115), *Malassezia globosa* (CBS 7966), *Aspergillus fumigatus* (AF293), and a set of viral genomes (RefSeq, 5 k + genomes). All reads that did not map exclusively to hg38 were defined as likely contamination and discarded from downstream processing. Sequence quality per sample was evaluated before as well as after trimming and decontamination using FASTQC 0.11.9 [20] and, in addition, all samples were analyzed as a collective with MULTIQC 1.9 [21]. The cleaned sample reads were aligned to the reference genome hg19 with bwa mem 0.7.17 [22] and duplicates were removed using picard 2.21.8 [23]. Samples that give alignments with less than 100 000 unique reads were rejected from the CNA‐analysis. For the following LowPass CNA‐profile analysis of the human genome was divided into non‐overlapping bins, each with a size of 500 kb. Mapped reads of the remaining samples were counted per bin, corrected, filtered, normalized, and segmented with the bioconductor package QDNAseq 1.26.0 8 [24]. The same package was used to create the log_2_(ratio) CNA‐profiles. The bioconductor packages ACE 1.8.0 [25] was employed to convert log_2_(ratio) into integer copy numbers.

### 2.9 Mutational panel sequencing

Genomic DNA from primary tumor, metastatic tissue, and tumoroids was extracted as described above. Fifty ng genomic DNA was sheared using the Covaris‐M220 (Covaris, Brighton, UK) (PIP(W): 75, Duty factor: 20%, Cycles per burst: 1000, Treatment time: 360 s, Target Peak: 170 bp) and used for library preparation. RNA samples were extracted using the MaxWell RSC RNA FFPE Kit (AS1440; Promega) according to the manufacturer's protocols. Fifty ng of both DNA and RNA were then used for library preparation.

The preparation of both RNA and DNA sample library were performed using the TSO500 Library Preparation Kit (TruSight Oncology 500 DNA/RNA Bundle, Cat. 20 028 216; Illumina®) strictly according to the TruSight Oncology 500 Reference Guide, document # 000000067621 v05. Then, a two‐step capture and enrichment of specific capture probes for DNA samples were performed. The DNA library and corresponding cDNA library of eight samples were normalized by using the library homogenization method based on magnetic bead purification and sequenced using the Illumina NextSeq 550 platform (Illumina). The Sequencing raw results were analyzed using the Container engine Docker/TruSight Oncology 500 v2.0 Local App (Nextseq) generating all data of SNV (single nucleotide variants), CNA, fusion transcripts, and splice variants. Data were then analyzed with the QIAGEN Clinical Insight (QCI) Interpret software (Qiagen).

### 2.10 RNA sequencing of tumoroids and bioinformatic analysis

Approximately 330 tumoroids with size around 100 μm (corresponds to approximately 100 000 cells) were harvested from Geltrex™ (Thermo Fisher Scientific) and lysed with RLT buffer (Qiagen) containing 1% β‐mercaptoethanol. RNA was isolated by using RNAeasy kit (Qiagen). Concentration and quality were determined by using Qubit and bioanalyzer. Only samples with RIN ≥8 were selected for RNA sequencing and sent to an external provider (CeGaT, Tübingen, Germany). After additional quality control, library preparation was performed by GeGaT using TruSeq Stranded mRNA (Illumina) library preparation kit. Libraries were sequenced on a NovaSeq 6000 (Illumina) with 2 × 100 bp QC values of sequencing.

Raw reads from three biological replicates (three bio banked tumoroid batches which are propagated independently) from tumoroid models were processed by the RNA‐Seq workflow of HIENA 0.9.5.7. Trimming, quality controls, and decontamination were performed analogously as described in the LowPass Whole Genome Sequencing (WGS) and CNA profiling section. Purified sample reads were aligned to the hg38 reference genome with STAR 2.5.1b [18]. Using featureCounts from Subread 2.0.0 [26], uniquely mapping exonic reads were counted per gene and per sample. Further quality criteria were evaluated, including library complexity (using Preseq 2.0.3 [20]), the genomic origin of the reads, and the 5′‐3′‐bias (both using QualiMap 2.2.2d [27]). The six samples' final counts table was utilized for differential expression analysis. The following steps were performed in R programming language 4.0.2. Gene raw counts, log (counts) as well as log (cpm), and top 500 differential expressed genes were visualized using Principle Component Analysis (PCA) and t‐distributed Stochastic Neighbor Embedding (t‐SNE) clustering techniques. For PCA, raw counts were scaled and prcomp function from stats package was employed. t‐SNE plots were constructed using Rtsne 0.1512 [28] as well as scater 1.14.6 [23]. For the differential expression analysis, we filtered out lowly expressed genes keeping only those having a minimum count of 10 per condition and a minimum total count of 15 across all samples. From the filtered count table, we obtained differentially expressed genes between the two models by applying the General Linear Modeling approach of the R‐package edgeR. [29]. All genes considered in the expression analysis were ranked decreasingly according to the formula $-\mathrm{lo}g_{10}\left(P\;\text{value}\right)\cdot \mathrm{sign}\left(\mathrm{lo}g_{2}\left(\mathrm{FC}\right)\right)$ before using them as input to KEGG pathway enrichment analysis which was achieved by Gene Set Enrichment Analysis (GSEA) using gseKEGG, from the R‐package clusterprofiler 3.18.1 [30]. From the results, terms of interest were selected manually. Enrichment plots were made using ggplot2 3.3.6 [31]. GSEA was also performed using the Hallmark gene sets of Molecular Signature Database (MSigDB). This was achieved using the GSEA function of clusterprofiler package.

### 2.11 Immunohistochemistry (tumoroids and FFPE tissue)

Tumoroids were harvested like already described above and fixed with 4% PFA at RT, overnight and embedded into the HistoGel (Thermo Fisher Scientific) according to the manufacturer's instructions. IHC stainings were performed on 2 μm thick FFPE sections as automated stainings according to the diagnostic standards of the institute of pathology on a Ventana BenchMark Ultra® (Roche, Basel, Switzerland). Heat‐induced epitope retrieval in Tris‐EDTA‐buffer or enzymatic antigen retrieval (Protease 1) was performed depending on the antibody used. Details on the antibodies, clones, dilutions, pretreatment conditions, and sources are listed in Table S1.

### 2.12 Flow cytometry

For characterization of surface proteins, tumoroids were dissociated to the single cell level using Cultrex Organoid Harvesting Solution (Bio‐Techne TrevigenBio). At least 200.000 cells per sample (1× antibody staining and 1 × isotype control staining) were resuspended in 100 μL of 1× PBS/2% FCS buffer and incubated with FcR Blocking Reagent (Miltenyi Biotec, Bergisch Gladbach, Germany) following surface marker staining for 20 min at RT prior to fixation and permeabilization (FluoroFix™ Buffer, Intracellular Staining Permeabilization Wash Buffer; Biolegend, San Diego, CA, USA). Antibodies against intracellular markers were added after permeabilization step and incubated for 30 min. The stained cells were washed and re‐suspended in 1× PBS/2% FCS buffer and analyzed by Kaluza software on a Gallios™ Flow Cytometer (Beckman Coulter). Antibodies and isotype controls are listed in Table S2.

### 2.13 Tumoroid drug screening

Tumoroid cultures from patient‐derived circulating tumor cells or breast cancer cell lines (MCF‐7 and BT‐474) were established according to Driehuis et al. [32]. Briefly, single cells were embedded in a basal matrix (Geltrex™ LDEV‐Free Reduced Growth Factor Basement Membrane Matrix; Thermo Fisher Scientific) at the density of 1.000–1.500 cells⋅μL^−1^ and seeded in 30 μL domes on standard culture plates. The plates were flipped upside‐down (during tumoroid expansion) and incubated at 37 °C for 40 min to allow polymerization of the matrix before adding culture medium. The composition of the culture medium is described in Section 2.5. The culture medium was exchanged every 3 days. Once the tumoroids reached a diameter > 100 μm, cells were dissociated using TrypLE™ Express (Thermo Fisher Scientific) and propagated as described above (2.5). CTC‐SGC‐01a and 01b are CTC‐derived tumoroids from the SGC patient No.1 (14 and 25 months after the diagnosis) and CTC‐SGC‐02 is CTC‐derived tumoroid culture established from SGC patient No.2 (56 months after the diagnosis).

For drug testing, cells were dissociated and seeded at 300 cells⋅μL^−1^ in 10 μL of Geltrex™ in 384 well μClear plates (Greiner Bio‐One, Kremsmünster, Austria). Tumoroids were allowed to form for 6 days, and growth was monitored by bright field microscopy. Three days after seeding, the media was exchanged using an Integra Viaflo 384 pipetting robot (Integra Biosciences, Zizers, Switzerland). For drug treatment, compounds (purchased from Selleckchem; Alpelisib and Lapatinib from AdooQ Bioscience; Tucatinib from MedChemExpress), antibodies (purchased from Selleckchem), and antibody‐drug conjugates (Trastuzumab‐Emtansine from university clinic hospital, Regensburg; Trastuzumab‐Deruxtecan was kindly provided by Daiichi Sankyo through an MTA) were prepared at the desired concentrations (Table S3) in culture medium in an intermediate 384 well plate (Greiner Bio‐One, Kremsmünster, Austria) and transferred to the assay plate using an Integra Viaflo 384 pipetting robot (Integra Biosciences, Zizers, Switzerland).

Tumoroid viability was measured 6 days after drug treatment using a CellTiterGlo‐3D® viability assay (Promega). Briefly, assay plates were equilibrated at room temperature for at least 30 min and CellTiterGlo‐3D® was added with a Multidrop™ Combi Reagent Dispenser (Thermo Fischer Scientific, Waltham, MA, USA). Plates were kept in the dark and shaken for 15 min on an orbital shaker at 200 rpm followed by 15 min incubation. Luminescence was measured using an EnVision® plate reader (PerkinElmer, Hamburg, Germany).

Viability data at different doses were normalized to the vehicle control and then used to fit a four‐parameter log‐logistic dose–response curve. Data processing and curve‐fitting were done using R (4.1.0) with R‐packages dplyr (1.0.8) and writexl (1.4.0) and an inhouse R‐package drl (0.1.1) depending on packages foreach (1.5.2) and doParallel (1.0.17).

### 2.14 Statistics

Statistical analysis was performed using the GraphPad Prism 8.0 software (GraphPad Software, Inc., Boston, MA, USA). All statistical steps for RNA‐Seq analysis were performed in R version 4.0.2 using native R packages and bioconductor packages. Dose–response curves and IC50 were calculated using an inhouse R package on R version 4.1.3.

## 3 Results

### 3.1 Generating CTC‐derived *in vitro* models from advanced stage cancer patients

**Fig. 1:**
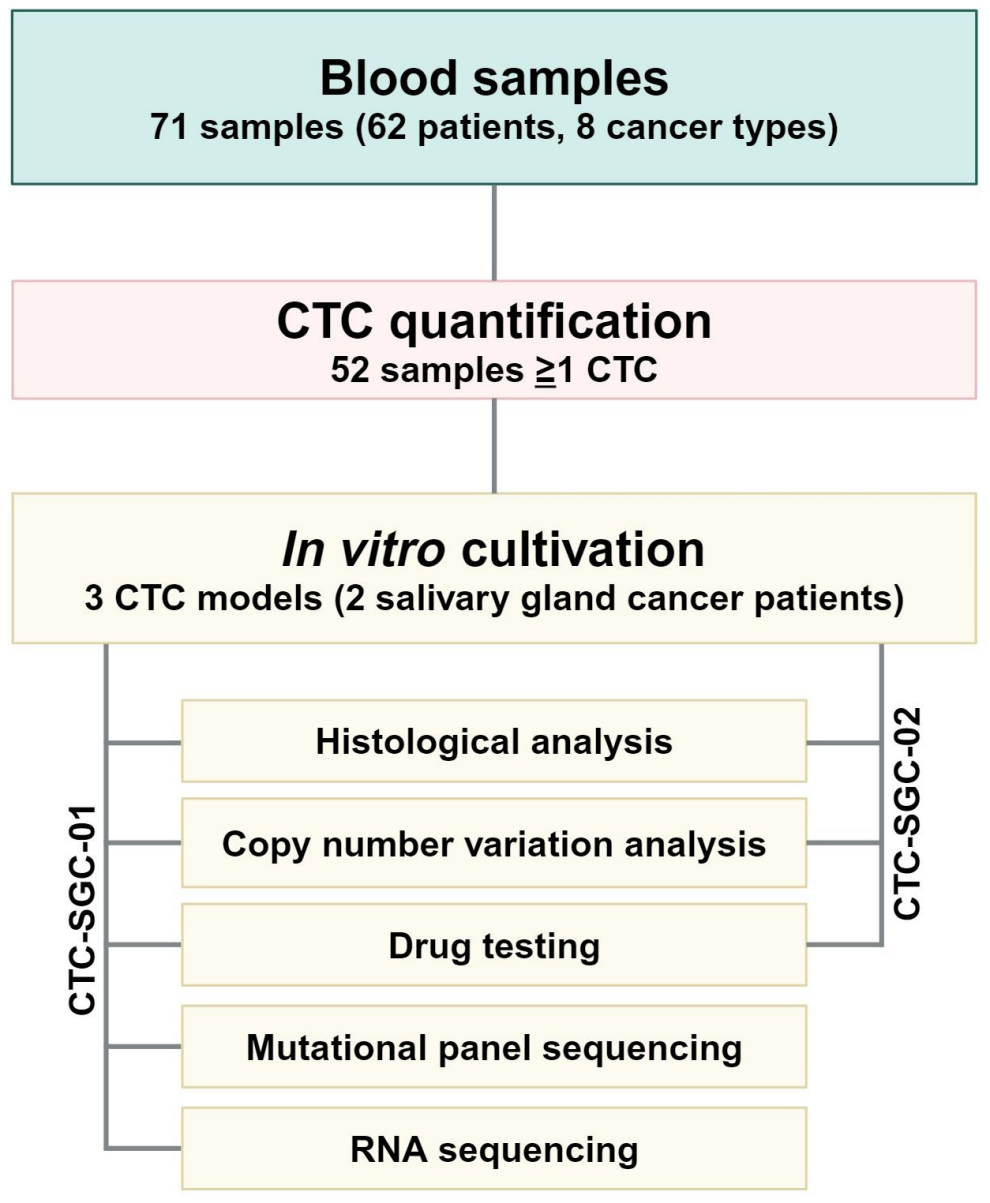
Study overview: To investigate if organoid culture condition support growth of CTCs, we screened 62 advanced cancer patients (71 samples) with 8 different cancer types for the presence of CTCs. All samples were subjected to organoid culture conditions and tumoroid growth was monitored. From 52 CTC‐positive samples only 3/3 SGC samples from 2 patients generated preclinical long‐term tumoroid models. Both models were further characterized by histological and copy number variation analysis as well as drug testing. For CTC‐SGC‐01, models derived from patient No.1 at 2 timepoints were additionally evaluated by mutational panel and RNA sequencing. CTC, circulating tumor cell; SGC, salivary gland cancer.

**Fig. 2:**
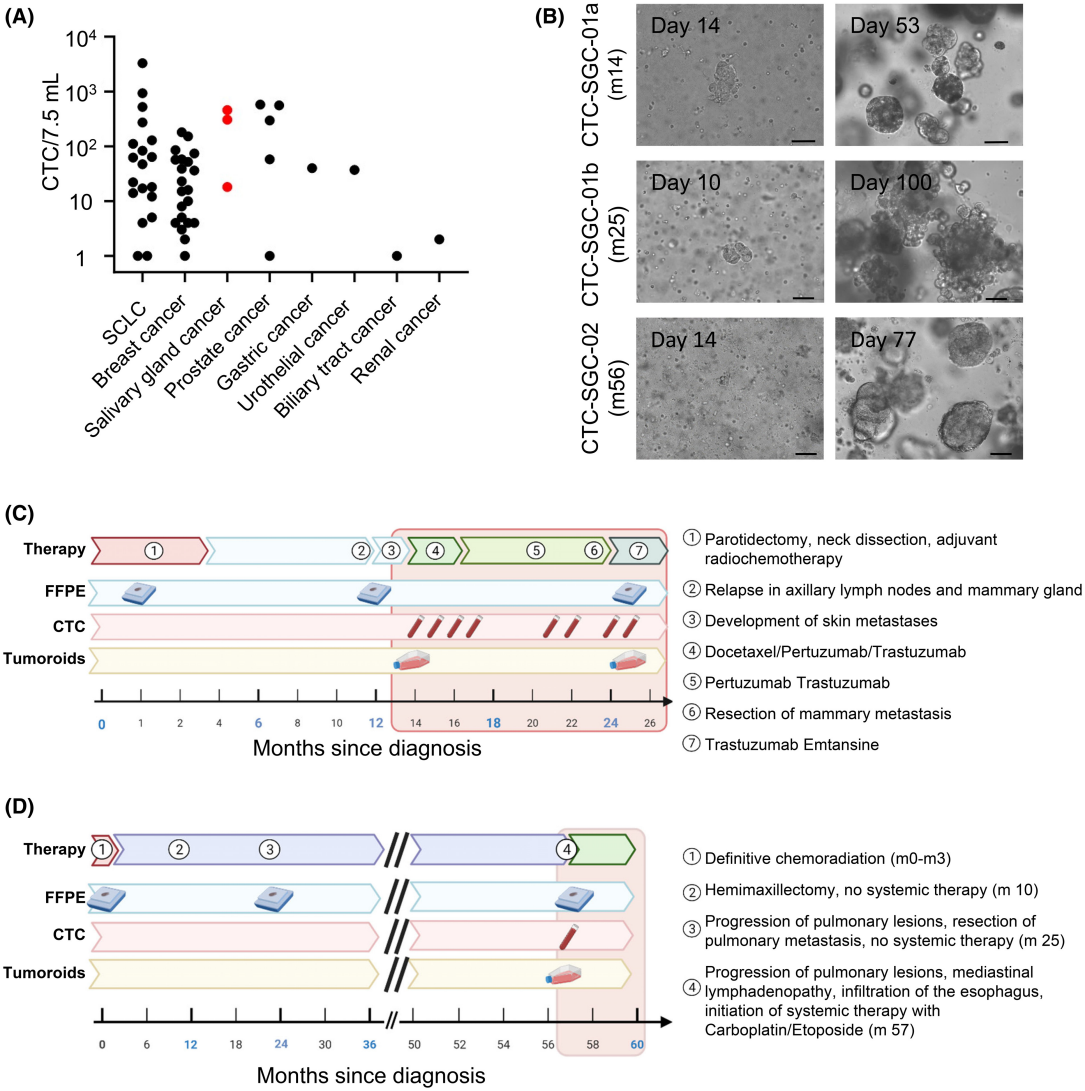
Generation of CTC‐derived models and course of disease for patient No.1 and No.2. (A) CTCs from various cancer patients (*n* = 71) were enumerated using the CellSearch® system and 52 patients with detectable CTCs are shown. CTCs were enriched by depletion of blood cells and subsequently cultured under organoid conditions. Successful generation of permanent models is marked as red dots (*χ* ^2^ test; *P* = 0.0005). (B) A phase contrast microscopy of tumoroid growth *in vitro* for both models of patient No.1 (CTC‐SGC‐01a and b) and No.2 (CTC‐SGC‐02). Scale bar represents 50 μm. (C) Course of disease of patient No.1. (D) Course of disease of patient No.2, row 1: duration of indicated therapies, row 2: routine histological specimens acquired at resection of primary tumor, lymph node biopsy at 1st relapse, and resection of a metastasis in the mammary gland at progression, row 3: blood draws for CTC measurement, row 4: time points of established CTC‐derived tumoroids. The red box indicates the treatment period in the oncology department. CTC, circulating tumor cell; FFPE, formalin‐fixed, paraffin‐embedded; SGC, salivary gland cancer; m, month; SCLC, small cell lung cancer.

In total, we enrolled 62 patients suffering from a variety of cancers (Table S4). Of these, CTCs were detected using the CellSearch® system in 52/71 blood samples, with CTC numbers ranging from one to 3292 (Figs 1 and 2A; Table S4). In order to generate *in vitro* models, additional EDTA blood containing viable cells was collected. CTCs were enriched by depletion of hematopoietic cells and subsequently cultured under organoid conditions as described [14]. Strikingly, although all patients suffered from late‐stage disease of evident aggressiveness, all CTC‐positive (and negative cultures) failed with two notable exceptions derived from two salivary gland cancer (SGC) patients that could be successfully expanded (3/3 blood samples, Figs 1 and 2A). Thus, from the 52 CTC‐positive samples obtained from eight cancer types only the three SGC samples successfully generated long term CTC‐derived models (*χ* ^2^ test; *P* = 0.0005; Fig. 2A).

The clinical course of the two patients is depicted in Fig. 2C,D. Briefly, patient No.1 was a 65‐year‐old patient presenting with an adenocarcinoma not otherwise specified of the right parotid gland. Initial treatment consisted of resection and combined radiochemotherapy in an adjuvant setting. Twelve months later the patient developed a new tumor mass in the right mammary region, axillary lymphadenopathy and multiple skin lesions on the upper right thorax, right cheek, and upper back accompanied by an erythema indicating aggressive metastatic disease. In this situation, the patient was referred to our oncology department for initiation of systemic therapy. Based on a significant HER2 overexpression in a lymph node (LN) biopsy (Fig. S1A), the patient was treated with combined immunochemotherapy with Docetaxel, Pertuzumab, and Trastuzumab. Despite an initial response the patient showed progression of the known mammary lesion, which was resected at month 23 and development of a new bone metastasis at month 25. The patient passed away shortly after initiation of Trastuzumab‐Emtansine as a second‐line therapy due to pneumonia. At month 14 (460 CTCs/7.5 mL) and 25 (305 CTCs/7.5 mL) we drew 9‐ and 12 mL of EDTA‐blood, respectively, depleted for hematopoietic cells and embedded the enriched CTCs in basal matrix. In both cultures, termed CTC‐SGC‐01a (month 14) and CTC‐SGC‐01b (month 25), initial tumoroids formed after 14 to 35 days and finally gave rise to 3D *in vitro* models. After 3–5 months of culturing, sufficient cell numbers for extensive model characterization and drug testing were available (e.g., for CTC‐SGC‐01a: 2.5 × 10^6^ cells after 105 days; Fig. 2B).

Patient No.2 had been diagnosed with adenoid cystic carcinoma (AdCC) 56 months before recruitment (Fig. 2D). Treatment included several surgical interventions and radiotherapeutic approaches in combination with Cisplatin chemotherapy. Eventually, 53 months after diagnosis, hepatic and pulmonary metastasis had progressed to a point where systemic therapy was indicated. Retrospectively, pulmonary metastasis was already evident at first diagnosis with a small nodule in the upper left lobe of the lung (Fig. S2B). At 56 months another biopsy from a pulmonary metastasis in the upper left lobe was acquired and showed a small cell neuroendocrine carcinoma (SCNEC) histology (Fig. S2A) and the patient was treated accordingly. Unfortunately, the patient rapidly progressed and passed away. CTC cultures of 7.5 mL blood at 56 months (18 CTCs/7.5 mL) showed initial tumoroids after 21 to 35 days that rapidly expanded to a 3D *in vitro* model (e.g., 2.8 × 10^6^ cells after 73 days) designated CTC‐SGC‐02 (Fig. 2B).

### 3.2 Histological analysis of CTC‐derived models

**Fig. 3:**
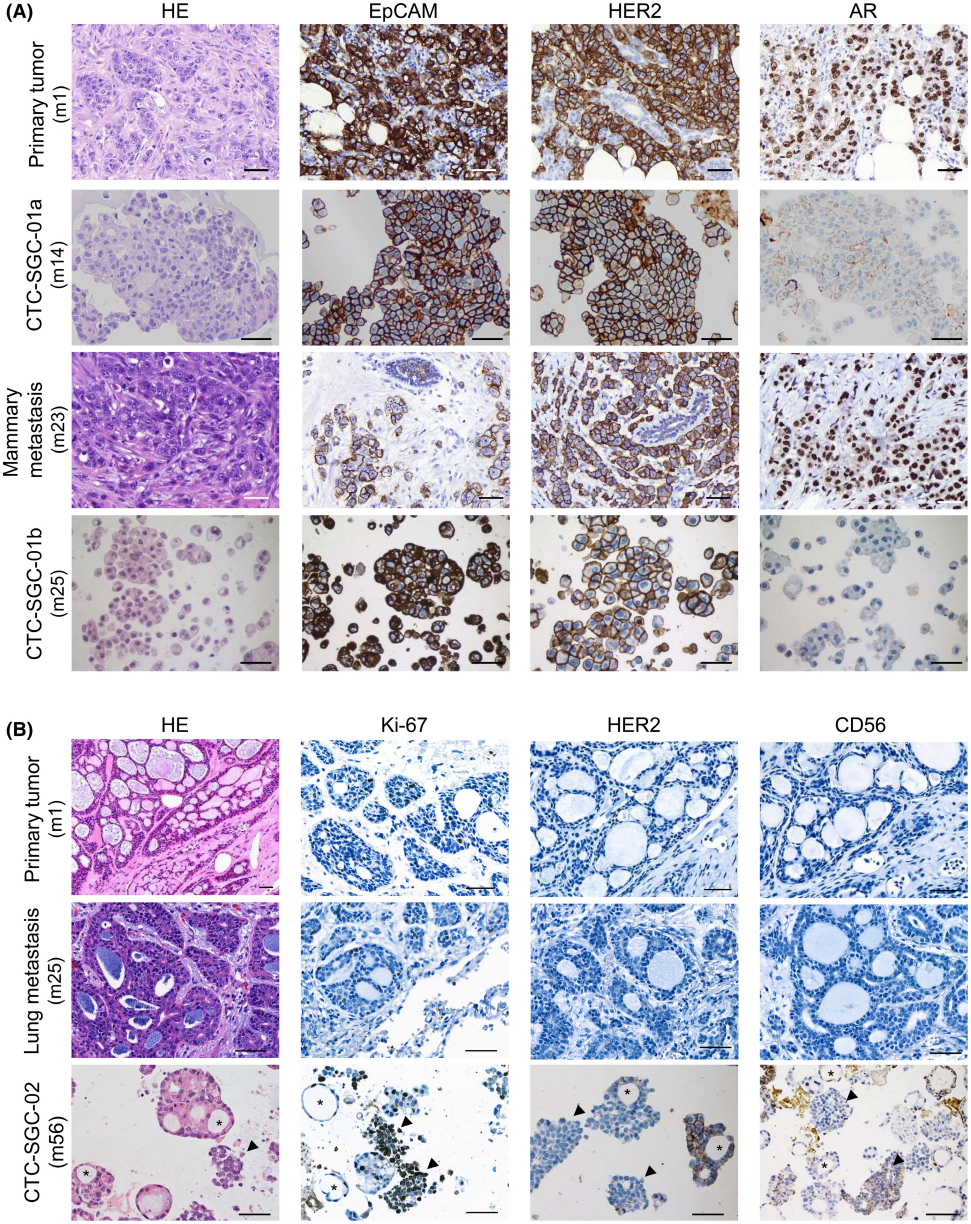
Immunohistochemical characterization of CTC‐derived models. (A, B) HE and IHC stains of primary tumors and tumoroid models from patient No.1 (A) and patient No.2 (B). Scale bar in all panels represents 50 μm; asterisks mark glandular, and triangles mark small cell‐like components. AR, androgen receptor; CTC, circulating tumor cell; HE, Hematoxylin Eosin stain; IHC, immunohistochemistry; m, month; SGC, salivary gland cancer.

We compared the tumoroid models with the corresponding FFPE tissue samples using histopathology and IHC. Both CTC‐derived tumoroid models from patient No.1 were morphologically and phenotypically similar to their corresponding primary tumor, lymph node, and breast metastasis, and to each other (Fig. 3A; Fig. S1A). Disparity was noted for androgen receptor expression (AR) that was present in all tissue samples but not detectable in CTC‐derived models which might therefore be explained by a selection during tumoroid culture (Fig. 3A, right columns). In contrast, EGFR was strongly expressed in both models and mammary metastasis, but not in the PT tissue samples which might indicate that CTC‐derived models representatively inform about the current disease status of the mammary metastasis regarding the EGFR expression (Fig. S1B). Despite the disease progression during anti‐HER2 treatment, all FFPE samples as well as both tumoroid models remained positive for HER2 (Dako Score 3+, Fig. 3A).

In patient No.2, the PT and lung metastasis at month 25 showed clear AdCC characteristics (Fig. 3B), while the developed tumoroid model revealed two cell populations that consisted of SCNEC cells expressing the neuroendocrine marker (CD56) and glandular tumoroids being positive for HER2 and EpCAM (Fig. 3B; Fig. S2C). In addition to IHC, we also analyzed the tumoroids by flow cytometry which confirmed the presence of two distinct CD56‐ or HER2‐expressing populations (Fig. S2D). Looking at Ki‐67 there was also a marked difference in proliferative activity between the glandular, CD56‐negative AdCC component and the SCNEC component of the tumoroids (Fig. 3B), the latter displaying higher Ki67‐positivity. The lung biopsy at month 57 showed mainly SCNEC histology with high proliferative activity (Fig. S2A).

In both cases the models largely reflected the histology of the patient's respective tumor and/ or metastasis tissue. However, minor disparities in terms of receptor expression such as EGFR and AR in patient Nr.1 as well as HER2 expression in patient Nr.2 could be observed.

### 3.3 Molecular analysis of CTC‐derived models

**Fig. 4:**
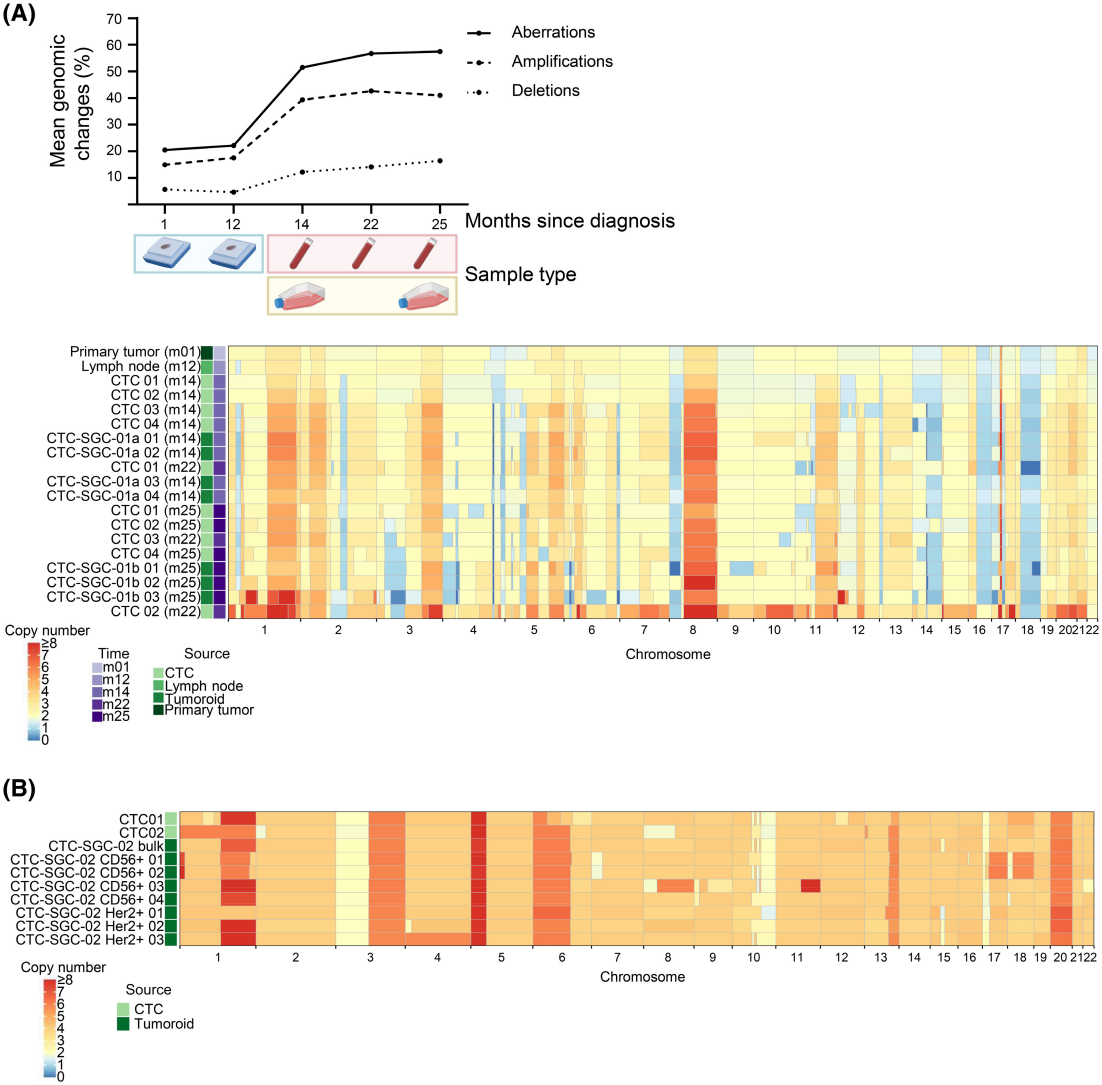
Analysis of copy number variations in FFPE tissue, single CTCs, and CTC‐derived models. (A) Upper Panel: Course of genomic changes (%) over time during the progression of the disease for the SGC patient No.1. The mean percentage of genome with overall aberrations, amplifications, and deletions is shown for indicated samples. Lower panel: CNA profiles of FFPE tissue, single CTCs, and single tumoroids generated at different months since diagnosis of patient No.1. (B) CNA profiles of two single CTCs, bulk DNA from tumoroids, and single HER2 or CD56 expressing tumoroids of patient No.2. CTC, circulating tumor cell; CNA, copy number alteration; FFPE, formalin‐fixed, paraffin‐embedded; m, month; SGC, salivary gland cancer.

In order to confirm the malignant origin of CTC‐derived models and to analyze clonal evolution, we performed shallow whole genome sequencing to assess copy number alterations (CNA). For patient No.1, we compared single CTCs before, during, and after anti‐HER2 therapy, single tumoroids of both models as well as PT and LN‐derived FFPE tissue. In general, CNA patterns of both CTC‐derived tumoroids were highly congruent to those of CTCs, LN metastasis, and PT with only minor genomic differences detected (Fig. 4A). However, the mean genomic copy number and the genome fragmentation (percentage of amplified/deleted genome) increased over the course of therapy and time (Fig. 4A; Table S5).

For patient No.2, we compared CNAs of two CTCs and CD56‐positive SCNEC cells as well as glandular HER2‐positive single tumoroids to discriminate whether the SCNEC cells were derived from an independent metachronous small cell lung cancer or if they were the result of near complete loss of differentiation of the adenoid cystic salivary gland carcinoma. The isolated single CTCs as well as the HER2‐ and CD56‐positive tumoroids displayed highly similar CNA profiles with characteristic gains (chromosomal arms 1q, 3q, 5p, 6p, 20p, 20q) and losses (chromosomal arms 3p, 10q, 17p) (relative to tetraploid) indicating a common molecular origin (Fig. 4B).

### 3.4 Drug screening to identify personalized treatments

**Fig. 5:**
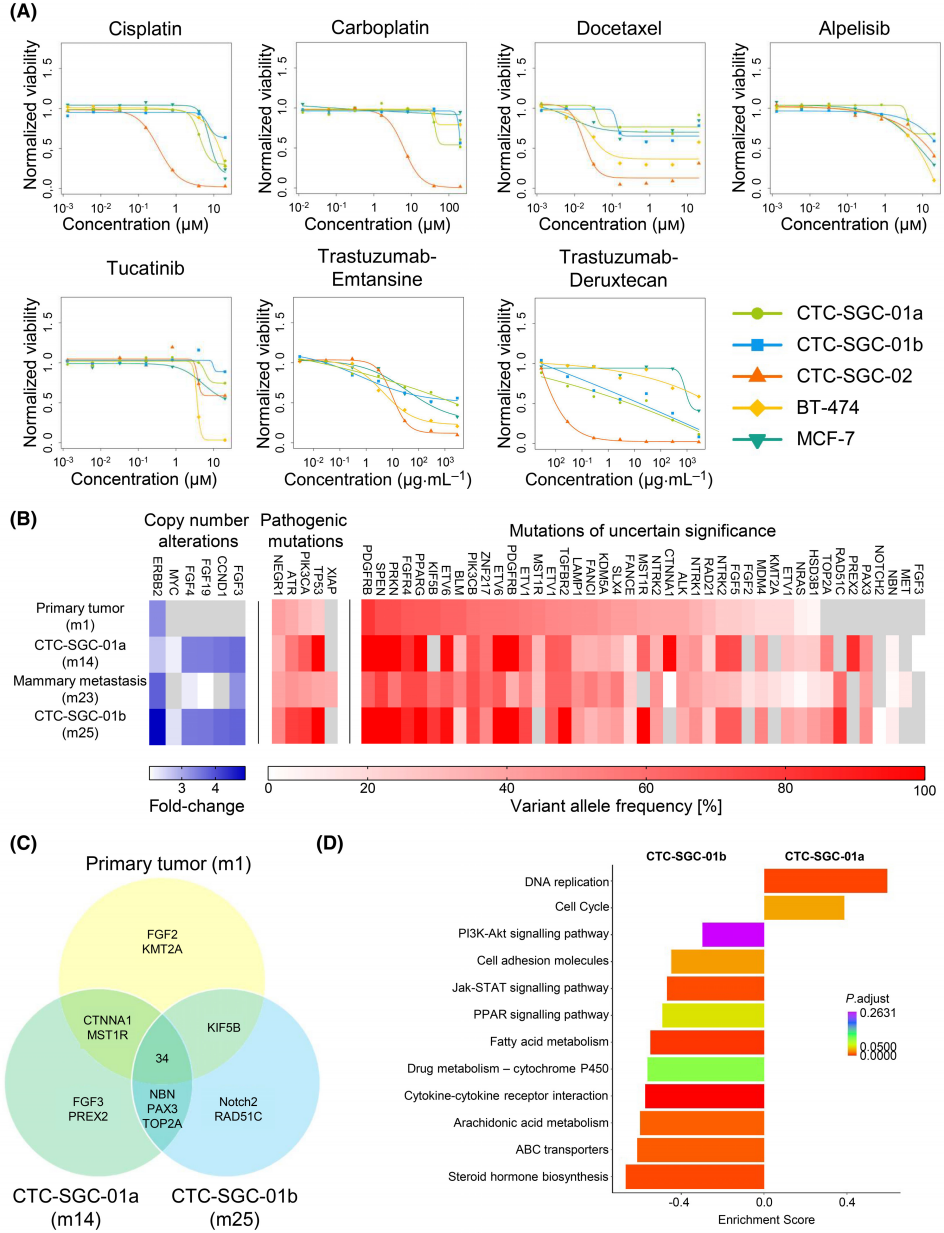
Drug screening and identification of potential resistance mechanisms by molecular characterization. (A) Patient‐derived tumoroid models (patient No.1: CTC‐SGC‐01a and CTC‐SGC‐01b; patient No.2: CTC‐SGC‐02) and control cell lines, BT‐474 (HER2‐positive) and MCF‐7 (HER2‐low/negative) were treated with Cisplatin, Carboplatin, Docetaxel, Alpelisib, Tucatinib, Trastuzumab‐Emtansine and Trastuzumab‐Deruxtecan. X‐axis has logarithmic scale. Concentrations for chemotherapeutics and small molecules are shown in μm and for antibody–drug conjugates in μg⋅mL^−1^. Panels display normalized dose–response curves for three independent experiments. (B) Mutations identified in the primary tumor, mammary metastasis, and tumoroids of patient No.1 by panel sequencing; (C) Shared mutations between primary tumor and both tumoroids of patient No.1; (D) Enrichment results for selected KEGG pathways computed by Gene Set Enrichment Analysis (GSEA) based on all differentially expressed genes. Enrichment score indicates the extent to which the differentially expressed genes are represented at the top or bottom of a ranked list of genes of the respective pathway (X‐axes). Y‐axis shows the KEGG terms of interest. Bar colors are based on the *P* adjust‐value of the KEGG term. Positive enrichment score indicates gene activation in CTC‐SGC‐01a and negative scores activation in CTC‐SGC‐01b. CTC, circulating tumor cell; KEGG, Kyoto Encyclopedia of Genes and Genomes; m, month; SGC, salivary gland cancer.

To identify candidate therapies for both patients we performed 3D tumoroid drug screening with a total of 13 compounds and three control reagents on both CTC tumoroid models of patient No.1 and the CTC tumoroid model of patient No.2 (Fig. 5A; Figs S3, S4). We used the HER2‐positive BT‐474 and the HER2‐low/negative MCF‐7 cell lines as controls.

CTC‐SGC‐02 tumoroids showed a high sensitivity to both platinum compounds and the chemotherapeutics Docetaxel and Epirubicin (Fig. 5A; Fig. S3, Table S6). The HER2‐ targeting antibody drug conjugate Trastuzumab‐Emtansine reduced the viability of tumoroids from patient No.2 to a similar extent as observed for the HER2‐positive BT‐474 cell line (Fig. 5A; Table S6). However, despite the heterogeneous HER2 expression, Trastuzumab‐Deruxtecan most efficiently killed CTC‐SGC‐02, indicating a superior effect of this antibody‐drug conjugate (Fig. 5A).

In contrast, both tumoroid models generated from patient No.1 were resistant to all tested chemotherapies and targeted drugs including Docetaxel which was part of the immunochemotherapy regimen administered between months 14 to 16 (Fig. 5A; Fig. S3). Only Trastuzumab‐Deruxtecan moderately reduced viability of CTC‐SGC‐01a and CTC‐SGC‐01b. In summary, whereas the CTC model from patient No.2 turned out to be sensitive to chemotherapeutics and HER2‐targeting therapies both CTC models from patient No.1 were apparently resistant to all tested chemotherapies as well as HER2‐targeting drugs. All three CTC‐derived models from both patients were resistant toward EGFR and FGF inhibitors as well as the combinatorial treatments (Fig. S4).

### 3.5 Identification of mechanisms of resistance or additional therapy targets

To gain first insight into potential resistance mechanisms of patient No.1 and to identify additional actionable molecular changes, we performed mutational panel sequencing (TruSight 500) and identified a total of 39 mutations within the PT, 41 mutations in CTC‐SGC‐01a, 44 mutations in the mammary lesion resected at 23 months, as well as 40 mutations in the CTC‐SGC‐01b tumoroid model (Fig. 5B; Table S7).

Thirty‐four mutations were shared by all samples and each sample displayed a few distinct alterations (Fig. 5C). Among the shared mutations that have been associated with primary drug resistance we identified TP53 and PIK3CA (Fig. 5B) [33, 34, 35]. In contrast, gene amplifications of FGF3, 4 and 19 only occurred in the metastasis as well as both models, indicating that FGF inhibitors might have been therapeutically effective [36]. However, FGF inhibitor Erdafitinib did not show an effect.

Finally, we performed RNA sequencing of both tumoroid models from the same patient (CTC‐SGC‐01a/b) established at 14 and 25 months. Only 528 (FDR <0.05 and log (FC) > 2) genes, among 16 067 genes that had non‐zero counts, showed a significant differential expression between both models (Table S8). Pathway enrichment on ranked 16 067 expressed genes with GSEA (Gene set enrichment analysis) revealed 45 significantly differentially regulated KEGG pathways with adjusted *P*‐value below 0.05 (Table S8) and selected pathways in Fig. 5D. Genes associated with fatty or arachidonic acid metabolism, cell adhesion molecules, JAK–STAT/PPAR signaling (enriched in CTC‐SGC‐01b) as well as cell cycle and DNA replication (enriched in CTC‐SGC‐01a) were significantly differentially enriched. Of note, we found an increased expression of genes associated with drug resistance, involved in PI3K signaling (not significant), and ABC transporters in the second sample CTC‐SGC‐01b (Fig. 5D; Table S8).

GSEA analyses with hallmark gene sets of MSigDB (Molecular Signature Database) revealed 13 differentially regulated hallmarks (Table S8, Fig. S5). Genes associated with Mitotic spindle, G2M checkpoint, MYC targets, DNA repair in CTC‐SGC‐01a and IL6 JAK–STAT3 signaling, Allograft rejection in CTC‐SGC‐01b, respectively, were significantly enriched. These results correspond nicely with the differentially regulated KEGG pathways (cell cycle, DNA replication in CTC‐SGC‐01a and JAK–STAT signaling in CTC‐SGC‐01b). In addition, we observe hallmark interferon gamma response, interferon alpha response, and inflammation response as significantly differentially enriched in CTC‐SGC‐01b.

### 3.6 CTC quantification and disease dynamics

**Fig. 6:**
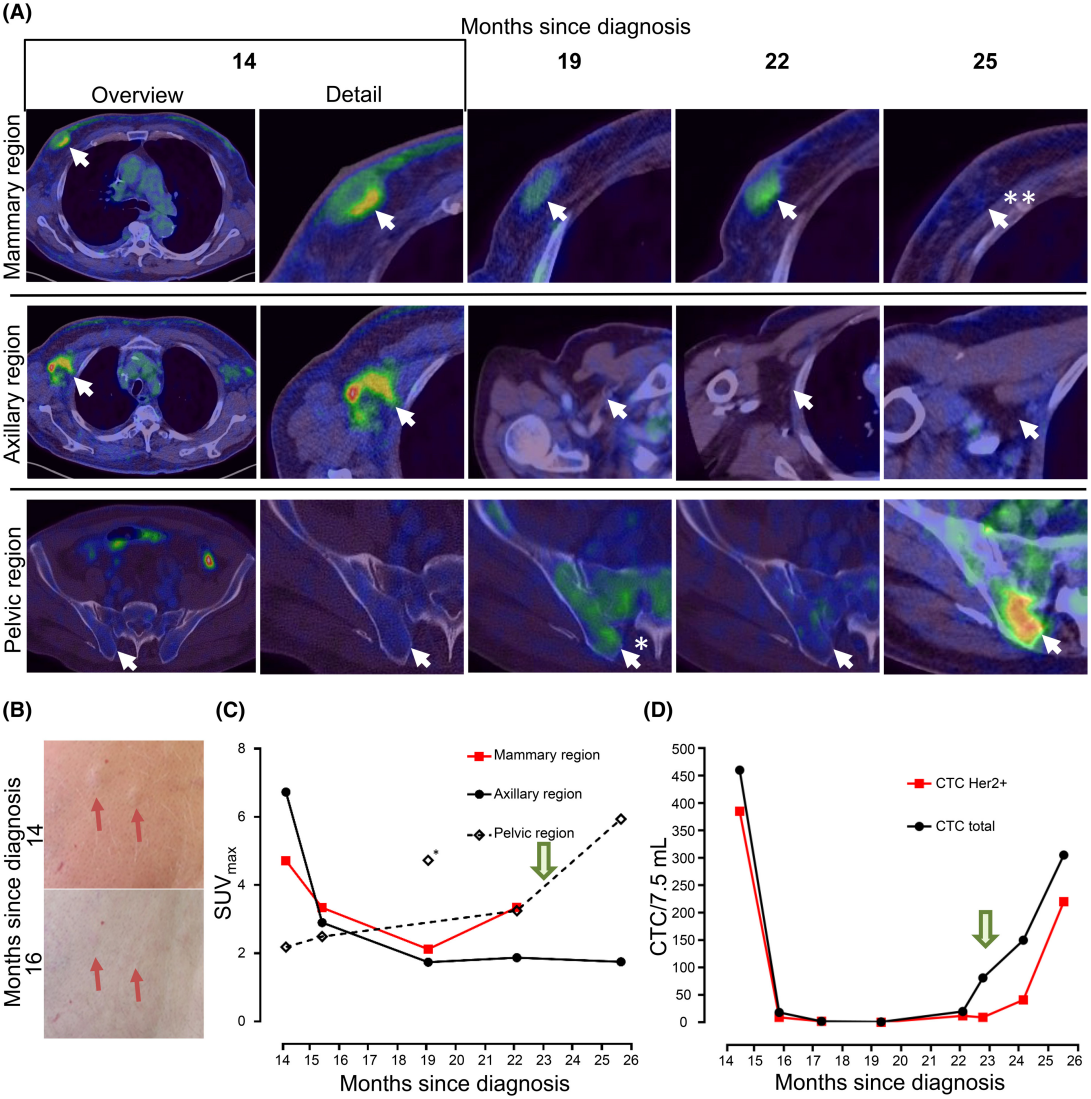
Case presentation, CTC quantification, and PET/CT imaging of patient No.1. (A) Representative PET/CT images from 1st relapse to month 25 at metastatic regions of impact (indicated by an arrow), * indicates high pelvic FDG‐uptake due to unspecific bone marrow activation under therapy, ** no standard uptake volume (SUV) measurable after complete resection of mammary metastasis. (B) *Upper panel*: skin metastasis at 1st relapse with intracutaneous nodule accompanied by skin erythema, *lower panel*: regression of skin metastasis 2 months after treatment initiation with Docetaxel, Pertuzumab, Trastuzumab. (C) Course of SUVmax in PET/CT imaging at metastatic regions of impact; * indicates high pelvic SUVmax most likely due to unspecific bone marrow activation under therapy. (D) Course of CTC counts quantified with the CellSearch® system in peripheral blood samples since first relapse; In C and D arrow indicates resection of mammary metastasis. CTC, circulating tumor cell; FDG PET/CT, fluorodeoxyglucose positron emission tomography/computerized tomography; SUV, standardized uptake value.

SGC‐CTC model‐based molecular characterization and drug testing could therefore inform about individual treatment options. Such information would be most useful if responses could be monitored. We therefore investigated if CTC counts can inform about disease progression. For patient No.1 we longitudinally quantified CTC levels including HER2 expression with the CellSearch® system (Menarini Silicon Biosystems) and compared the results to routine tumor imaging by Positron Emission Tomography – Computed Tomography (PET/CT). At month 14 we detected 460 CTCs/7.5 mL (62% of CTCs expressing HER2), indicative of the presence of mammary, axillary, and multiple skin lesions (Fig. 6A–D). After initiation of combined immunochemotherapy the skin lesions had completely regressed (Fig. 6B) and PET/CT confirmed treatment response of the LN‐ and the mammary metastasis (Fig. 6A,C). Similarly, CTC counts rapidly dropped to only one HER2‐negative CTC/7.5 mL blood at month 19. The next staging performed at month 22 (7 months after systemic treatment initiation) showed progression of the known mammary lesion, while all other lesions were still in remission (Fig. 6A,C). In parallel, CTC count increased again to 81 CTCs/7.5 mL with only 11% of CTCs being positive for HER2 indicating a potential resistance to the HER2 targeted therapy. The mammary metastasis was then successfully resected and although there was no clinical evidence for further progression yet, CTC counts rose further to 150 CTCs/7.5 mL blood (27% HER2 positive) at month 24 and 305 CTCs/7.5 mL (72% HER2 positive) at month 25 (Fig. 6A,C,D). Restaging PET/CT at month 25 confirmed development of new bone metastases (Fig. 6A,C).

## 4 Discussion

Therapeutic progress for rare cancers like SGC remains difficult to achieve as conducting adequately powered trials is hardly feasible. For these patients, molecular tumor boards have been established that try to exploit additional molecular information. However, direct identification of drug sensitivities by functional *in vitro* testing may provide an additional source of information, especially for patients with rare cancers for whom the standard of care is inadequately defined and the interpretation of molecular data is hampered by low numbers of patients.

Our data suggests that CTCs from advanced salivary gland cancers seem to be particularly prone to grow in cultures in striking contrast to all other cancer entities tested, although several of them displayed high CTC numbers. We further show that the CTC‐derived cultures closely reflect the histology and molecular features of uncultured *ex vivo* analyzed samples. Notably, the generation of models that could be used in small‐scale drug screens took less than 3 months, which can be sufficient to enable generation of treatment relevant information for many patients. However, larger studies are needed to systematically investigate the efficacy of CTC expansion in SGC as well as the correlation of *in vitro* drug vulnerabilities with patient responses.

Since cfLB is useful for monitoring the total tumor load, the focus of our study was to identify which cancer (sub)types might benefit from cLB, specifically by the ability of CTCs to grow in culture. We compared 8 cancer types, of which at least SCLC, breast, and prostate cancer had previously been successfully expanded in rare occasions [8, 37, 38, 39, 40, 41, 42, 43, 44, 45]. Here, only SGC samples formed tumoroids which were molecularly and functionally analyzed. So far, only two studies reported on CTC assessment of only 18 patients with AdCC and neither of them tested expansion in culture [46, 47]. Therefore, a systematic investigation of SGC CTC numbers and tumoroid formation in future studies is needed. Notably, for patient No.1 we compared at the same blood draw time point CTC numbers in peripheral vs. central vein blood using a port catheter system and detected many more cells in the central vein blood sample. This single data point is consistent with the pioneering study of Crosbie et al. in lung cancer, in which the positivity rate doubled in central vein samples [48]. Thus, future clinical study protocols should consider central vein sampling in order to facilitate CTC detection and model generation.

Although our functional and molecular analyses could not be used for clinical guidance in the two cases depicted above, our study demonstrates the potential of cLB to guide precision oncology decision making if performed under appropriate regulatory conditions. In patient No.1, we identified genomic features fully compatible with primary therapy resistance against various classes of drugs; however, genomic changes acquired over time clearly indicated adaptive mechanisms in response to treatment. Among the targeted therapies, only Trastuzumab‐Deruxtecan showed moderate efficacy *in vitro*; however, gene expression profiles also pointed towards changes in PI3K‐pathway activation and ABC transporters that have been associated with resistance to HER2‐targeting therapies or chemotherapies [49, 50]. The identified FGF3, 4, and 19 gene amplifications, indicated another potential vulnerability; however, *in vitro* testing also revealed a resistance against FGF inhibitors. Still, larger‐scale drug screens using all available cancer drugs might have identified suspected and unexpected treatment options. In patient No.2, our small‐scale drug testing identified multiple drugs effective *in vitro*. Unfortunately, this patient's disease was already too advanced for their implementation.

Interestingly, the model generated from patient No.2 also reflected the clonal dynamics of the patient's heterogeneous disease. An invasive biopsy would have been necessary to exclude the growth of an SCLC in addition to the SGC. Both the glandular and the small cell endocrine morphology were found in generated tumoroids and molecular genetics confirmed a common origin. Since the SCNEC cells became the disease drivers, a drug screen would include this population.

## 5 Conclusion

Our data suggest that prospective studies should be performed to explore the unique chances provided by cLB specifically in the context of SGC. CTCs in this cancer type not only provide quantitative information about treatment response and molecular information for DNA‐based target prediction but most importantly are prerequisite for cellular expansion *in vitro* for functional drug screening within reasonable time frames. Although we need a further study to more accurately understand how well CTC‐derived models represent the current patient situation, two analyzed cases open the perspective for further investigation of the translational potential of CTC‐derived tumoroid models in cancer treatment decisions in SGC. It further suggests that systematic cLB screenings in other rare cancer subtypes may be also worth exploring. Such protocols could be included in basket/umbrella studies embedded in comprehensive precision oncology networks that are large enough to recruit appropriate numbers of patients.

## Conflict of interest

The authors declare no conflict of interest.

## Author contributions

FL, CW, NS, DaH, TP, BP, and CAK contributed to the conceptualization. NS, FL, CW, DaH, BP, KH, and CAK contributed to the methodology. NS, FL, DaH, ST, GF, AC, TS, ARV, XL, KW, CB, SMR, FK, KE, FW, WD, MH, JW, CW; JG, DiH, MA, UK, CS, ASL, PU, SS, and RM contributed to the investigation. FL, CW, NS, FK, MH, and JG contributed to the visualization. CW, BP, TP, and CAK contributed to the funding acquisition. FL and CW contributed to the project administration. TP, CW, DaH, and CAK contributed to the supervision. CW, FL, DaH, NS, and CAK contributed to the writing—original draft. All authors contributed to the writing—review and editing.

## Peer review

The peer review history for this article is available at https://www.webofscience.com/api/gateway/wos/peer‐review/10.1002/1878‐0261.13741.

## Supporting information


**Fig. S1.** Microscopic characterization of primary tumor, metastatic lesions, and CTC‐derived tumoroids of patient No.1.
**Fig. S2.** Clinical, microscopic and flow cytometry characterization of patient No.2.
**Fig. S3.** Drug sensitivity testing on patient derived tumoroids.
**Fig. S4.** EGFR and FGF blockade in patient derived tumoroids.
**Fig. S5.** Enrichment results for selected Hallmark gene sets from MSigDB computed by Gene Set Enrichment Analysis (GSEA).

**Table S1.** Antibodies used for immunohistochemistry.

**Table S2.** Antibodies and isotypes used in flow cytometry.

**Table S3.** Drugs and concentrations.

**Table S4.** Patient information.

**Table S5.** Quantification of copy number alterations.

**Table S6.** Tumoroid and control cell line drug tests: AUC, Emax, IC50.

**Table S7.** Overlapping mutations in patient No.1.

**Table S8.** RNA‐Seq pathway analysis for both models of patient No.1 and differential expressed genes.

## Data Availability

The data that support the findings of this study are available on request from the corresponding author. Patient data are not publicly available due to privacy or ethical restrictions.
