# Supplementary material for: Cellular liquid biopsy provides unique chances for disease monitoring, preclinical model generation and therapy adjustment in rare salivary gland cancer patients
Source: Mol Oncol. 2024 Oct 5;19(7):2056–73. doi: 10.1002/1878-0261.13741 (PMC12234380; doi:10.1002/1878-0261.13741)
Supplement: Supplementary file 1 — Fig. S1. Microscopic characterization of primary tumor, metastatic lesions, and CTC‐derived tumoroids of patient No.1. Fig. S2. Clinical, microscopic and flow cytometry characterization of patient No.2. Fig. S3. Drug sensitivity testing on patient derived tumoroids. Fig. S4. EGFR and FGF blockade in patient derived tumoroids. Fig. S5. Enrichment results for selected Hallmark gene sets from MSigDB computed by Gene Set Enrichment Analysis (GSEA). [file MOL2-19-2056-s007.pdf]

Cellular liquid biopsy provides unique chances for disease monitoring, preclinical model generation and therapy adjustment in rare salivary gland cancer patients

Nataša Stojanović Gužvić, Florian Lüke, Steffi Treitschke, Andrea Coluccio, Martin Hoffmann, Giancarlo Feliciello, Adithi Ravikumar Varadarajan, Xin Lu, Kathrin Weidele, Catherine Botteron, Silvia Materna–Reichelt, Felix Keil, Katja Evert, Florian Weber, Thomas Schamberger, Michael Althammer, Jirka Grosse, Dirk Hellwig, Christian Schulz, Stephan Seitz, Peter Ugocsai, Anke Schlenska-Lange, Roman Mayr, Ulrich Kaiser, Wolfgang Dietmaier, Bernhard Polzer, Jens Warfsmann, Kamran Honarnejad, Tobias Pukrop, Daniel Heudobler, Christoph A. Klein and Christian Werno

Supplementary figures

Figure S1. Microscopic characterization of primary tumor, metastatic lesions, and CTC-derived tumoroids of patient No.1.

Figure S2. Clinical, microscopic and flow cytometry characterization of patient No.2.

Figure S3. Drug sensitivity testing on patient derived tumoroids.

Figure S4. EGFR and FGF blockade in patient derived tumoroids.

Figure S5. Enrichment results for selected Hallmark gene sets from MSigDB computed by Gene Set Enrichment Analysis (GSEA).

Figure S1

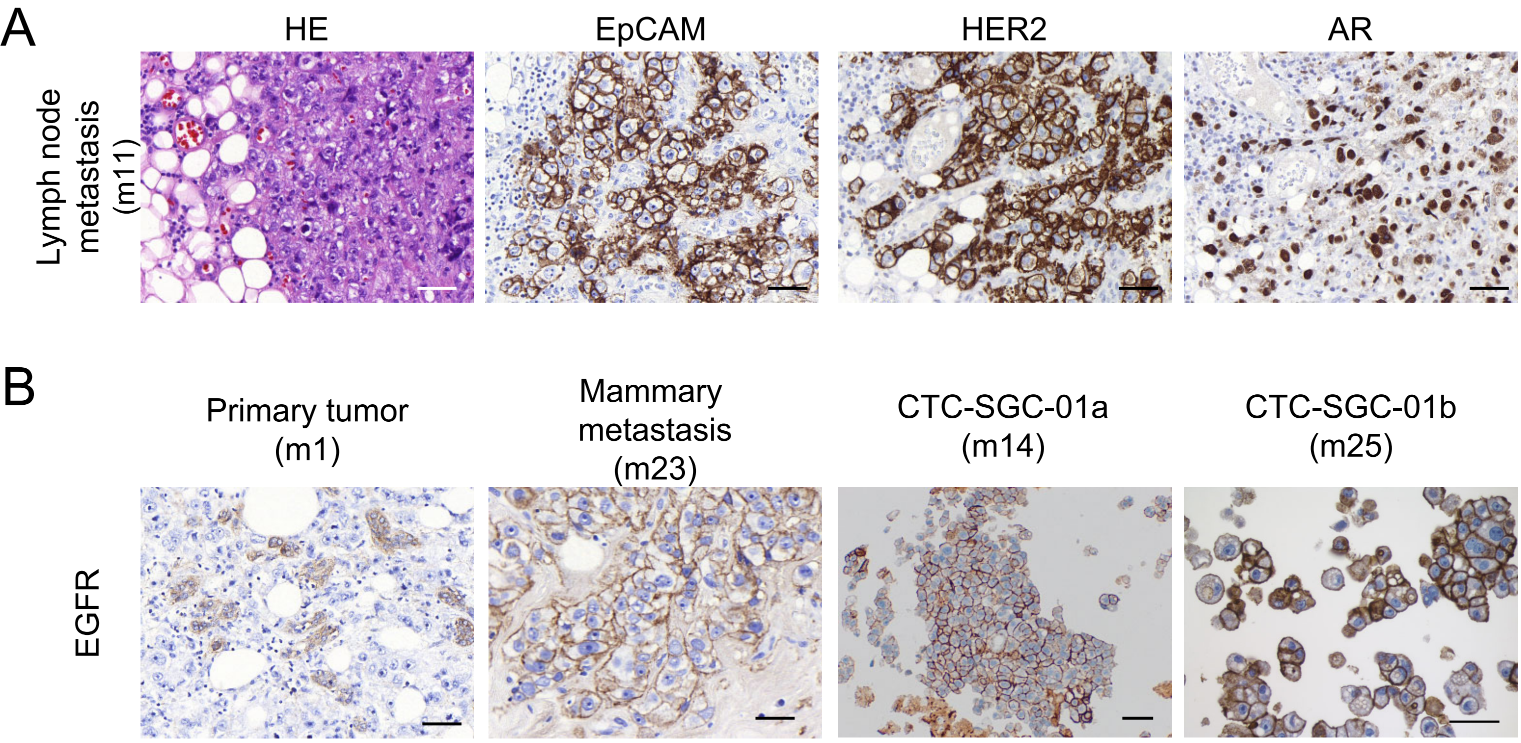

Figure S1. Microscopic characterization of primary tumor, metastatic lesions, and CTC-derived tumoroids of patient No.1. A: HE and IHC stains for EpCAM, HER2, and AR of a lymph node metastasis of patient No.1; **B:** EGFR expression in primary tumor, mammary metastasis, and both patient (No.1) derived tumoroids. Scale bar in all panels represents 50 µm; HE: Hematoxylin Eosin stain, AR: androgen receptor, m: month.

Figure S2

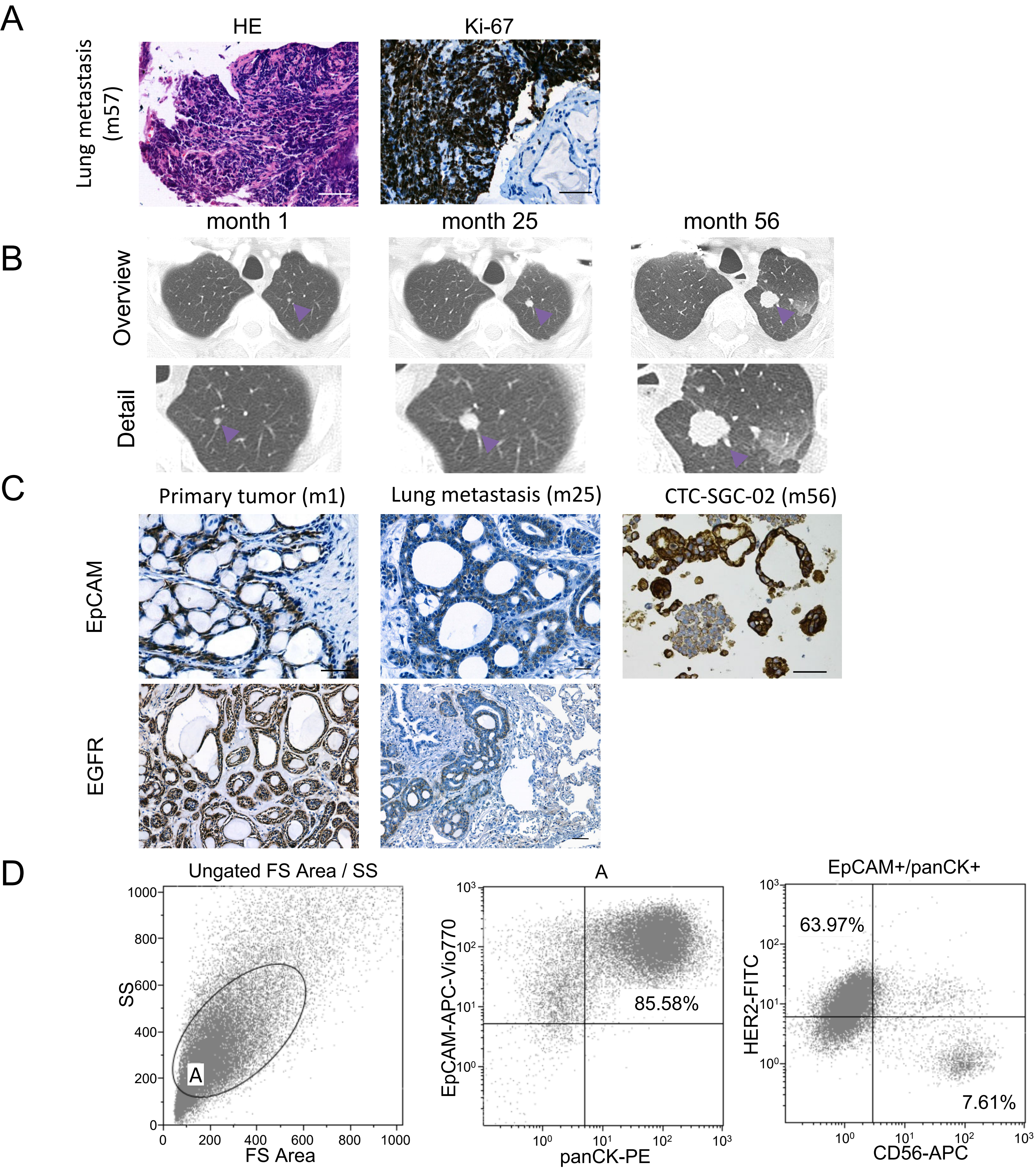

A
HE
Ki-67
Lung metastasis (m57)
month 1
month 25
month 56
B
Overview
Detail
C
Primary tumor (m1)
Lung metastasis (m25)
CTC-SGC-02 (m56)
EpCAM
EGFR
D
Ungated FS Area / SS
SS
FS Area
A
EpCAM-APC-Vio770
panCK-PE
85.58%
EpCAM+/panCK+
HER2-FITC
CD56-APC
63.97%
7.61%

Figure S2

Figure S2. Clinical, microscopic and flow cytometry characterization of patient No.2.
A: HE and IHC stains for Ki-67 of patient No.2’s lung metastasis at 57 months since diagnosis. Scale bar represents 50 µm; **B:** **L**ongitudinal thoracic computed tomography imaging from first diagnosis to month 56 showing progression of an initially small pulmonary nodule over the course of the disease, growth rate appears to be increasing strongly from month 53 to month 56 indicating dedifferentiation; **C:** EGFR and EpCAM expression in primary tumor, lung metastasis and, tumoroids of patient No.2; **D:** Flow cytometric analysis of tumoroid derived single cell suspension. Left panel: gating strategy in side and forward scatter. Middle panel: 85.58 % of gated cells (A) are positive for EpCAM and panCK. Right panel: expression of HER2 and CD56 on EpCAM+/panCK+ cells.

Figure S3

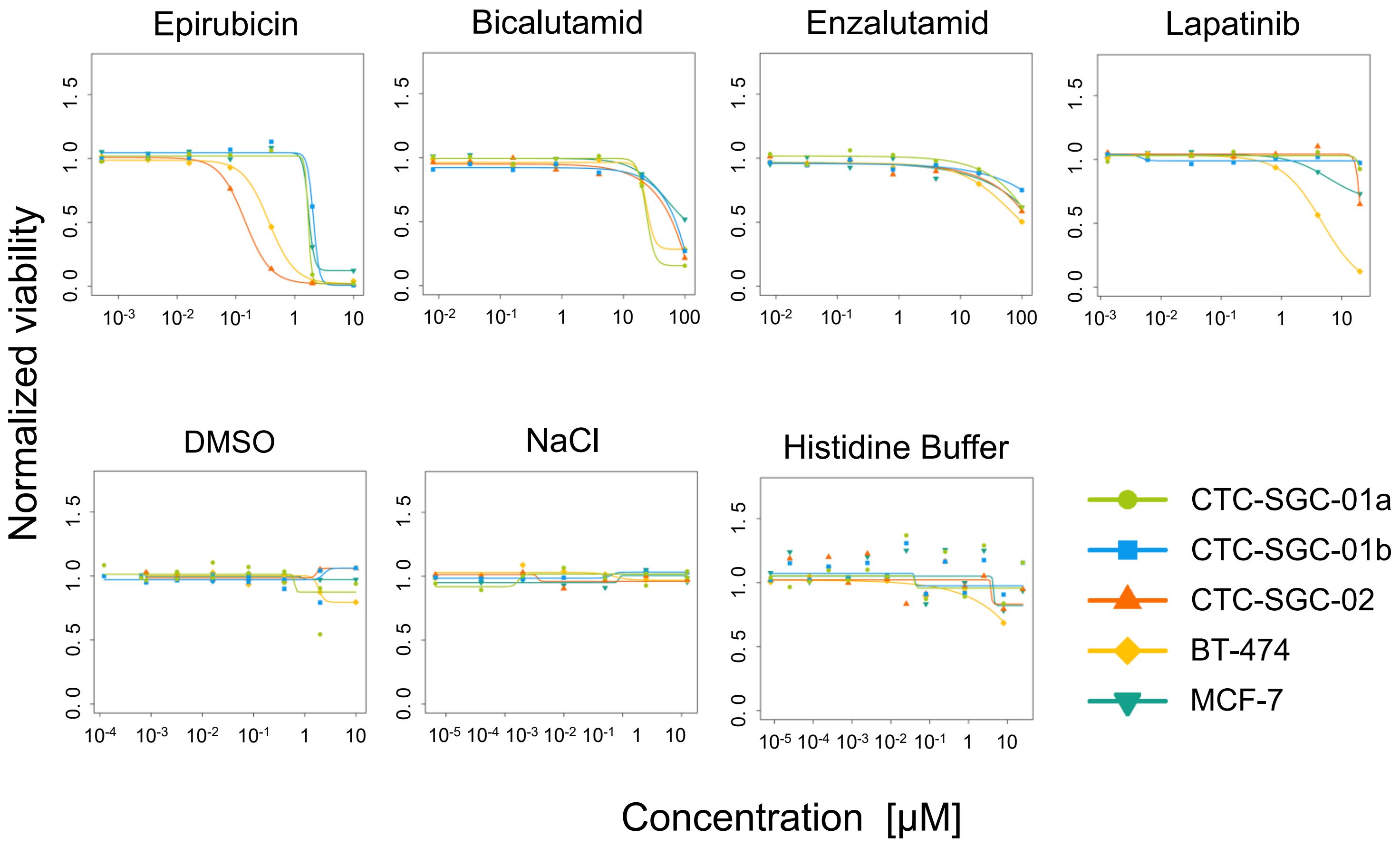

Figure S3. Drug sensitivity testing on patient derived tumoroids. Patient derived tumoroid models (patient No.1: CTC-SGC-01a and CTC-SGC-01b; patient No.2: CTC-SGC-02) and control cell lines, BT474 (HER2-positive) and MCF7 (HER2-low/negative) were treated with Epirubicin, Bicalutamid, Enzalutamid, Lapatinib. Dose response curves are also shown for the vehicle controls (DMSO, NaCl and Histidine Buffer). X-axes have logarithmic scale. Concentrations are shown as μM. Panels display normalised dose response curves for 3 independent experiments generated with R-script.

Figure S4

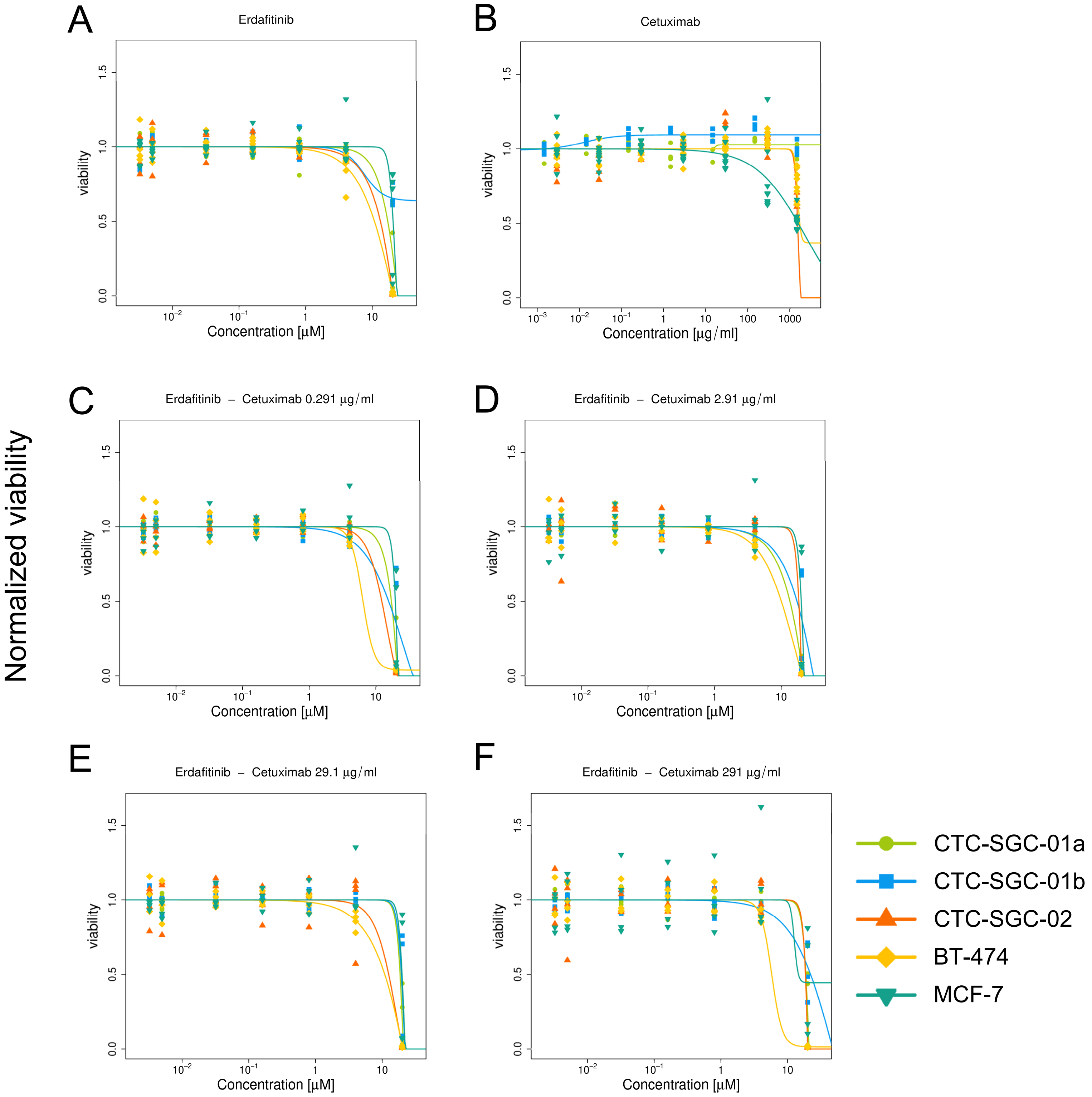

Figure S4. EGFR and FGF blockade in patient derived tumoroids. Patient derived tumoroid models (patient No.1: CTC-SGC-01a and CTC-SGC-01b; patient No.2: CTC-SGC-02) and control cell lines, BT474 (HER2-positive) and MCF7 (HER2-low/negative) were treated with **A:** Erdafitinib and **B:** Cetuximab only as well as with combination of constant concentration for Cetuximab **C:** 0.291 µg/ml; **D:** 2.91 µg/ml; **E:** 29.1 µg/ml; **F:** 291 µg/ml) and dose for Erdafitinib. X-axes have logarithmic scale. Concentrations are shown as µM or as µg/ml for Cetuximab. Panels display normalised dose response curves for 3 technical replicates and 2 independent experiments, generated with R-script.

Figure S5

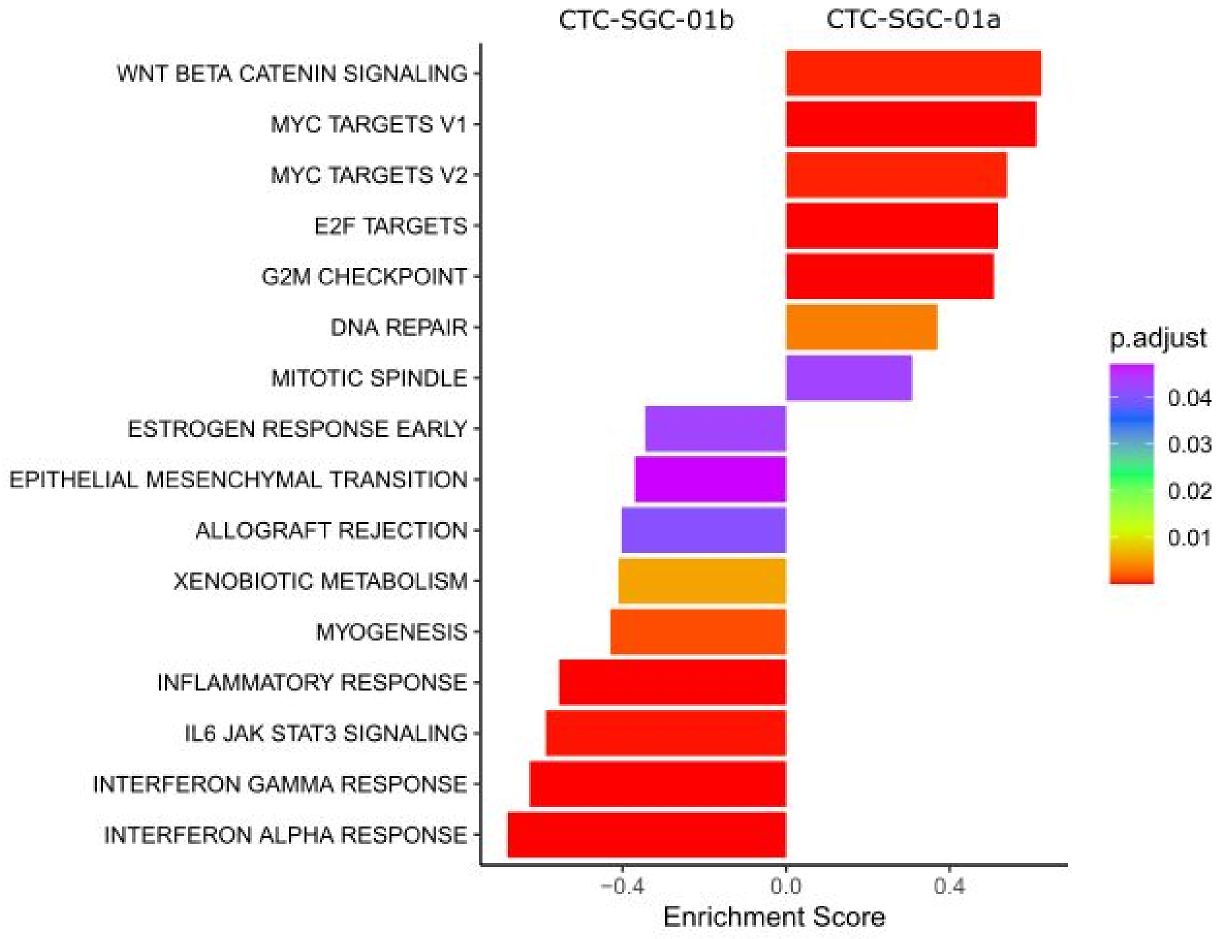

Figure S5. Enrichment results for selected Hallmark gene sets from MSigDB computed by Gene Set Enrichment Analysis (GSEA). GSEA was performed on all expressed genes. Enrichment score indicates the extent to which the differentially expressed genes are represented at the top or bottom of a ranked list of genes of the respective hallmark gene set (X-axes). Y-axis shows the hallmark terms of interest. Bar colors are based on the p.adjust-value of the hallmark gene set term. Positive enrichment score indicates gene activation in CTC-SGC-01a and negative scores activation in CTC-SGC-01b.
